# The Nucleus/Mitochondria-Shuttling LncRNAs Function as New Epigenetic Regulators of Mitophagy in Cancer

**DOI:** 10.3389/fcell.2021.699621

**Published:** 2021-09-08

**Authors:** Yan Li, Wei Li, Andrew R. Hoffman, Jiuwei Cui, Ji-Fan Hu

**Affiliations:** ^1^Key Laboratory of Organ Regeneration and Transplantation of Ministry of Education, Cancer Center, First Hospital of Jilin University, Changchun, China; ^2^Stanford University Medical School, VA Palo Alto Health Care System, Palo Alto, CA, United States

**Keywords:** mitochondria, mitophagy, cancer metabolism, long non-coding RNA, cancer stem cells, cancer therapy

## Abstract

Mitophagy is a specialized autophagic pathway responsible for the selective removal of damaged or dysfunctional mitochondria by targeting them to the autophagosome in order to maintain mitochondria quality. The role of mitophagy in tumorigenesis has been conflicting, with the process both supporting tumor cell survival and promoting cell death. Cancer cells may utilize the mitophagy pathway to augment their metabolic requirements and resistance to cell death, thereby leading to increased cell proliferation and invasiveness. This review highlights major regulatory pathways of mitophagy involved in cancer. In particular, we summarize recent progress regarding how nuclear-encoded long non-coding RNAs (lncRNAs) function as novel epigenetic players in the mitochondria of cancer cells, affecting the malignant behavior of tumors by regulating mitophagy. Finally, we discuss the potential application of regulating mitophagy as a new target for cancer therapy.

## Introduction

Mitochondria play a central role in cellular bioenergetics, regulating essential biochemical reactions that generate adenosine triphosphate (ATP) and reactive oxygen species (ROS; Friedman and Nunnari, 2014; Spinelli and Haigis, 2018; Missiroli et al., 2020). Abnormalities in respiratory chain structure or mutations of mitochondrial genome DNA can interfere with normal mitochondrial functions, leading to an imbalance in calcium ion homeostasis, ROS production, and apoptosis. Mitochondria quality control (MQC) is a critical mechanism to maintain mitochondrial health and mitochondrial homeostasis (Pickles et al., 2018), including mitochondria biogenesis, fission and fusion, and mitophagy (Wai and Langer, 2016). Mitophagy, a specialized form of autophagy that degrades dysfunctional mitochondria, serves as the primary mechanism to regulate mitochondrial functions, including energy metabolism, metabolic reprogramming, and mitochondria self-repair and renewal (Pickles et al., 2018; Roca-Portoles and Tait, 2021). Mitophagy is activated by hypoxia, metabolic stress, and mitochondrial depolarization through regulatory pathways involving PINK1/Parkin, BNIP3/NIX, and FUNDC1 (Poole and Macleod, 2021).

**FIGURE 1:**
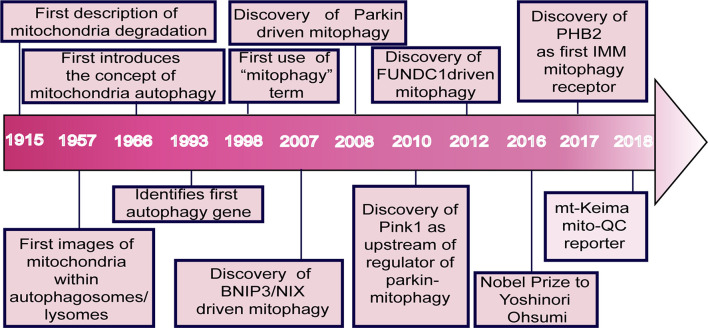
Major events in the field of mitophagy. The idea of mitochondrial degradation was first suggested by Lewis and Margaret in 1915. With the use of electron microscopy, the presence of mitochondria within vesicles and lysosomes in rat tissues was observed in 1957. de Duve postulated the concept of cellular autophagy in 1966. Ohsumi’s lab identified the first autophagy gene in 1993 (Tsukada and Ohsumi, 1993). In 1998, the term mitophagy was first used by Klionsky and Scott (Scott and Klionsky, 1998). BNIP3L was shown to play a vital role in mitophagy during mammalian erythrocyte differentiation in 2007 (Schweers et al., 2007). Parkin and PINK1 were characterized in 2008 (Narendra et al., 2008) and 2010 (Matsuda et al., 2010), respectively. Chen et al. discovered the mitophagy receptor FUNDC1 in 2012. Ohsumi won the Nobel Prize for his groundbreaking work in the field of autophagy in 2016. Wei et al. (2017) found the IMM protein, prohibitin 2 (PHB2), as a crucial mitophagy receptor in 2017. In 2018, Juliette et al. used a fluorescent pH-biosensor system to generate the mito-QC reporter for assessing basal mitophagy *in vitro* and *in vivo* (Lee J. J. et al., 2018). A fluorescent probe system called mt-Keima was also developed, which uses a pH-sensitive protein with a pH-dependent shift in fluorescence excitation to assess mitophagy in cells and tissues.

The concept of mitochondrial degradation was first proposed by Margaret and Warren Lewis as early as 1915 (Lewis and Lewis, 1915). Later studies using electron microscopy demonstrated the presence of mitochondria within lysosomes (Clark, 1957). However, this morphological observation was not followed up with functional experiments until the landmark studies of Okamoto et al. (2009), who identified ATG32 as a critical receptor of mitophagy in yeast, a finding which won the Nobel Prize in 2016. Since then, studies on mitophagy have dramatically increased (Figure 1; Montava-Garriga and Ganley, 2020).

The role of mitophagy in tumorigenesis has recently been studied in detail. Since mitophagy leads to the degradation of dysfunctional mitochondria and decreased ROS production, mitophagy was initially thought to be related to tumor suppression. However, it has been shown that mitophagy actually plays a complicated role in tumor growth. Mitophagy is vital for metabolic remodeling within tumor cells and for modulating interactions among tumor cells, but its role is complex and depends on the type and stage of the tumor.

Recent studies have shown that lncRNAs, a class of long non-coding RNA (lncRNA) molecules with a length of >200 bp, have emerged as critical regulators in cancer. Many lncRNAs are transcribed from the human genome, and a number of them are involved in the regulation of mitophagy and mitophagy-associated drug resistance. LncRNAs can regulate various cellular functions, including mitochondrial metabolic reprogramming, to meet the needs of tumor metabolism. Therefore, it is important to understand the relationship between lncRNAs and mitophagy regulation in cancer. Exploiting the newly emerging knowledge of the lncRNA-mitophagy-cancer axis may provide novel targets for cancer therapy. In this review, we will summarize recent progress on the role of lncRNAs in malignancy, demonstrating the importance of mitochondria-associated lncRNAs in cancer metabolism, apoptosis, and mitophagy (Dong et al., 2017; Zhao et al., 2018; Anand and Pandi, 2021).

## The Role of Mitophagy in Cancer

### Regulatory Pathways of Mitophagy

The main physiological function of mitophagy is to ensure the recycling of old and damaged organelles through continuous mitochondrial housekeeping (Palikaras et al., 2018). Little is known about steady-state mitophagy levels (also known as basal mitophagy) as mitophagy is typically examined under stress-induced conditions. Cells undergo basal mitophagy as part of routine mitochondrial maintenance (McWilliams et al., 2016). In tissues that consume large amounts of ATP, such as the brain, skeletal muscle, heart, liver and kidney, mitochondrial biogenesis is actively maintained to meet the cells’ metabolic requirements. When cells are switched from an energy-consuming state to a stable state, they tend to activate mitophagy in order to reduce mitochondrial mass as an adaptation to changes in cell metabolism. Proper homeostasis of mitochondrial mass is therefore critical to maintaining biological resilience (Onishi and Okamoto, 2021). Mitophagy is also induced during cell fate determination, such as erythrocyte differentiation (Schweers et al., 2007; Sandoval et al., 2008), cardiomyocyte maturation (Gong et al., 2015; Gottlieb and Bernstein, 2015; Kuznetsov et al., 2020; Morales et al., 2020), the developmental transitions of muscle tissue (Sin et al., 2016), and stem cell pluripotency (Vazquez-Martin et al., 2016; Xiang et al., 2017). In addition, mitophagy regulates the elimination of sperm mitochondria, thereby avoiding the inheritance of paternal mitochondrial DNA (mtDNA; Rojansky et al., 2016; Song et al., 2016). Since mitophagy plays a crucial role in maintaining mitochondrial homeostasis (Bin-Umer et al., 2014), it is not surprising that defective, inadequate, or excessive mitophagy can result in pathological conditions (Palikaras et al., 2017), such as neurodegenerative disease (Um and Yun, 2017; Tran and Reddy, 2020; Wang et al., 2021), cardiovascular disease (Billia et al., 2011; Zhang W. et al., 2016; Zhang et al., 2017; Chang et al., 2020), metabolic disorders (He et al., 2021; Pang et al., 2021; Zia et al., 2021), inflammation (Sliter et al., 2018; Lee et al., 2020), liver disease (Ke, 2020; Kouroumalis et al., 2021), aging (Richard et al., 2013; Cornelissen et al., 2018), and cancer.

**FIGURE 2:**
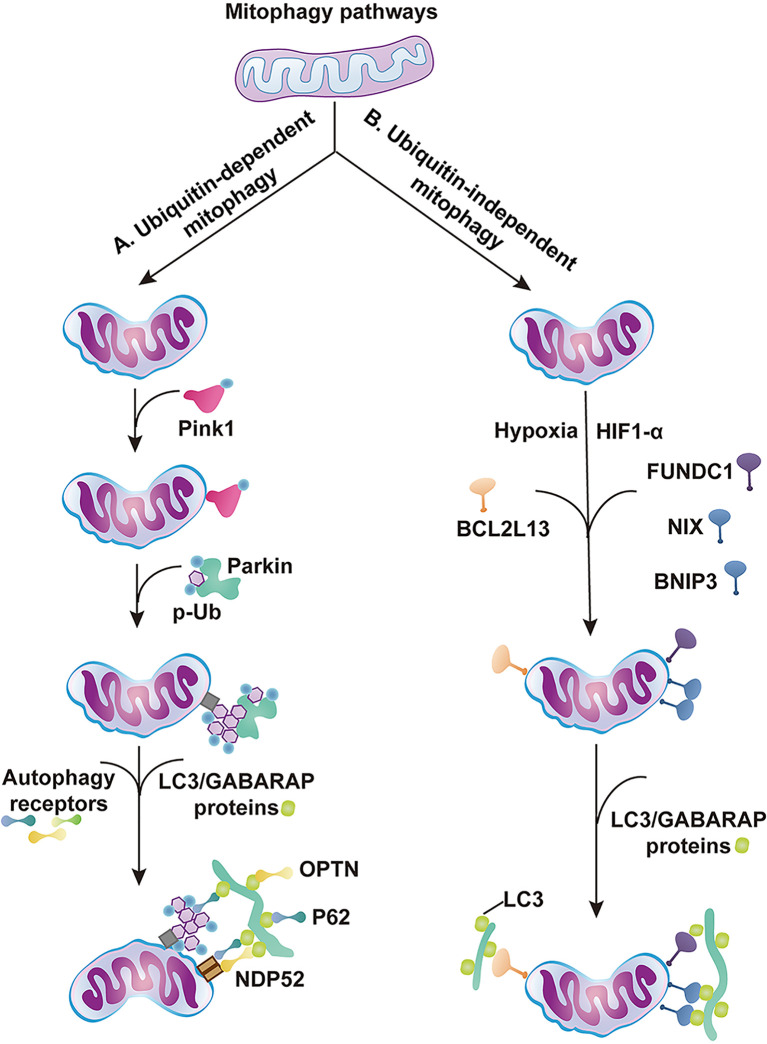
Major regulatory pathways of mitophagy. The schematic diagram summarizes the ubiquitin (Ub)-dependent and independent mitophagy pathways. **(A)** Ub-dependent mitophagy. (1) Aberrations in mitochondrial membrane potential leads to stabilization and activation of PINK1 on OMM. (2) The PINK1 kinase phosphorylates basal Ub and the cytosolic Ub ligase Parkin. Parkin is recruited to the mitochondria and activated by phospho-Ub and PINK1 phosphorylation. (3) Generation of Ub chains recruit mitophagy receptors, such as OPTN, NDP52, or p62. (4) Autophagosome machinery is recruited by mitophagy receptors. Mitophagy receptors then interact with LC3/GABARAP proteins coating the forming phagophore. **(B)** Ub-independent mitophagy. (1) Hypoxia stimulates HIF1α expression, and FUNDC1, NIX, and BNIP3 accumulate on the OMM and interact with LC3 to mediate mitophagy. (2) BCL2L13 interacts with LC3 proteins to mediate mitophagy (Murakawa et al., 2015).

Mitophagy is induced during stress to mediate metabolic adjustments to an external challenge. Mitophagy is highly regulated by various signaling pathways that can be roughly classified as ubiquitin (Ub)-dependent and Ub-independent mitophagy. The PINK1/Parkin pathway relies on a Ub-dependent mitophagy mechanism (Greene et al., 2012; Yamano and Youle, 2013; Harper et al., 2018). After PINK1-mediated phosphorylation, the E3 ligase PARKIN is translocated to mitochondria, where it induces the ubiquitination of the outer membrane of mitochondria. The mitophagy cargo adaptors p62, OPTN, and NDP52 bind to the phospho-Ub chains and interact with processed LC3 to target mitochondria for phagosome degradation (Figure 2A; Trempe et al., 2013; Kazlauskaite et al., 2014; Koyano et al., 2014). The inner mitochondrial membrane (IMM) protein PHB2 is a crucial mitophagy receptor that is required for Parkin-induced mitophagy (Wei et al., 2017; De Falco et al., 2020). BNIP3, BNIP3-like (BNIP3L) or NIX proteins and FUNDC1, on the other hand, function via Ub-independent mitophagy (Schweers et al., 2007; Liu et al., 2012; Wu et al., 2014). After hypoxia or other stress stimulation, BNIP3 and NIX can form stable homodimers in the outer mitochondrial membrane and recruit LC3 to induce mitophagy (Figure 2B; Rogov et al., 2017; Marinković et al., 2021; Springer et al., 2021). Both pathways cooperate to ensure efficient mitophagy (Lee et al., 2011; Zhang T. et al., 2016).

### Mitophagy as a Positive Promoter of Tumorigenesis

The PINK1/Parkin pathway activates mitophagy in a Ub-dependent manner in cancer. As compared with non-transgenic controls, Parkin-knockout (KO) mice exhibited a significantly lower tumor burden of melanoma, including tumor size and lung metastases (Lee Y. S. et al., 2018). PINK1 was upregulated in esophageal squamous cell carcinoma (ESCC) tissues from patients who received chemotherapy (Yamashita et al., 2017). ESCC patients with high CD44 expression were more likely to have distant metastases and poor prognosis. Interestingly, mitophagy is increased in CD44 high-expressing cells (Whelan et al., 2017). Depletion of Parkin or inhibition of autophagy with chloroquine reduced CD44 high-expressing cells both *in vitro* and *in vivo*. Collectively, these studies suggest that PINK1/Parkin-dependent mitophagy may play an essential role in promoting the development of tumors.

BNIP3 plays a pro-tumorigenic role by driving aggressive features in melanoma cells, including migration, clonal growth, cell survival, and vasculogenic mimicry (Maes et al., 2014). Knockdown of baseline BNIP3 in melanoma cells caused accumulation of actin stress fibers and membrane ruffles and induced aberrations in cytoskeletal structures. Thus, BNIP3 maintains the plasticity of the actin cytoskeleton, accounting for cell migration and cancer progression. By analyzing immunohistochemical scores, Jiang et al. (2018) found that elevated expression of BNIP3 was found in 31.9% (15/47) of patients with uveal melanoma. High expression of BNIP3 was associated with deeper scleral invasion and lower cancer survival. These studies suggest that BNIP3/NIX-dependent mitophagy may play a pro-tumorigenic role in the development of cancer.

FUNDC1 also plays a critical role in hypoxia-induced mitophagy. Hui et al. (2019) found that FUNDC1 was upregulated in tumor tissues of laryngeal cancer patients in parallel with lipid peroxidation. Treatment of laryngeal cancer cells with low doses of hydrogen peroxide upregulated FUNDC1 through the ERK1/2 signal pathway and promoted proliferation of laryngeal cancer cells. Similarly, FUNDC1 was also upregulated in cervical cancer tissues, and the FUNDC1 expression score was an independent factor to determine overall and disease-free survival. Knockdown of FUNDC1 reduced the proliferation of cervical cancer cells and enhanced cell sensitivity to cisplatin and radiotherapy (Hou et al., 2017).

**FIGURE 3:**
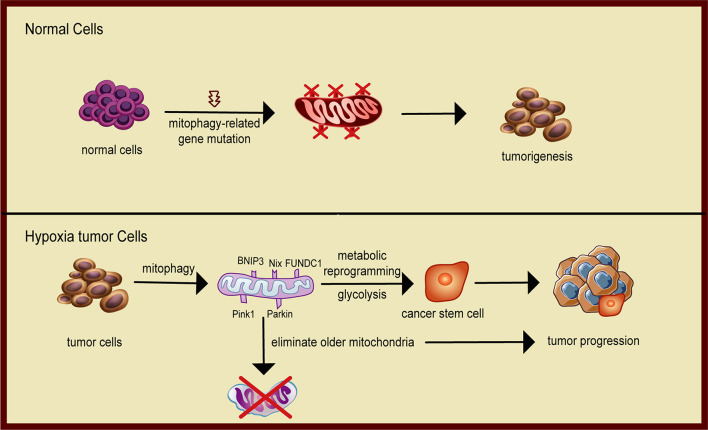
Schematic diagram of mitophagy promoting tumor progression. In normal cells, mitophagy eliminates damaged mitochondria and protects the normal metabolism of the cells. When mitophagy is inhibited, cells are prone to carcinogenesis. In solid tumors, cancer cells face metabolic stress from limited oxygen and nutrient availability. Mitophagy removes dysfunctional mitochondria that cannot metabolically handle the nutrient stress and maintain the cancer stem cell phenotype, hence facilitating tumor progression.

Recent studies have shown that mitophagy plays an important role in the metabolic transition of tumors and the maintenance of phenotype of cancer stem cells (CSCs). Continuous reprogramming of cellular metabolism is a critical milestone event in cancer (Pavlova and Thompson, 2016; Chen et al., 2020; Ohshima and Morii, 2021). Metabolic alterations lead to enhanced uptake and utilization of glucose and amino acid nutrients from a nutrient-deficient environment and increased use of glycolysis/TCA cycle intermediates for macromolecular biosynthesis. Cancer cell metabolism is governed by the Warburg effect: aerobic glycolysis with the increased rate of glycolysis and lactate production despite exposure to ambient oxygen. It is now clear that mitophagy is an important cellular mechanism that facilitates the metabolic switch to a glycolytic phenotype (Naik et al., 2019). Both canonical and non-canonical mitophagy contribute to alterations of bioenergetics and the metabolome, enhancing cellular developmental capability. Metabolic transformation of tumor cells, such as increased aerobic glycolysis or reduced oxidative phosphorylation, is controlled by altered expression and activity of several key enzymes involved in metabolism (Pavlova and Thompson, 2016). Mitophagy may play a critical role in this reprogramming by eliminating dysfunctional and damaged mitochondria. The turnover of mitochondria activates mitochondrial biogenesis to generate new mitochondrial mass that more effectively responds to nutrient stress (Drake et al., 2017). Hypoxia-inducible factor 1 (HIF-1) is a key factor that coordinates mitophagy and mitochondria turnover by regulating gene expression. In response to chronic hypoxia, HIF-1 initiates mitophagy by regulating the expression of mitophagy receptors BNIP3 and BNIP3L. The reduction of mitochondrial mass decreases total oxygen consumption of tumor cells and enhances cell survival under hypoxic stress (Vara-Perez et al., 2019; Figure 3).

Mitophagy is also required for self-renewal and maintenance of stemness (Figure 3). CSCs exhibit enhanced potential for metastatic spread and contribute to the resistance to anticancer therapies. CSCs have unique metabolic signatures and heavily depend on the coordination of mitochondrial oxidative phosphorylation and mitophagy to maintain CSC phenotypes, including glycolytic reprogramming, epithelial-to-mesenchymal transition, survival after chemotherapeutic challenge, and increased formation of ROS (Sessions and Kashatus, 2021). Liu et al. (2017) reported that mitophagy was a positive regulator of hepatic CSCs. Active p53 binds to the promoter of NANOG and prevents OCT4 and SOX2 transcription factors from activating that stemness gene, leading to a reduction in hepatoma stem cells. When mitophagy is activated, p53 is phosphorylated at serine-392 by PINK1, a mitophagy-associated kinase, and is degraded by a mitophagy-dependent mechanism. Thus, mitophagy maintains hepatic CSCs by targeting the activities of tumor suppressor p53. Katajisto et al. (2015) found that mitophagy may play a critical role in regulating stemness by isolating and eliminating old mitochondria during asymmetric division of stem cells. Collectively, these studies demonstrate that mitophagy plays a positive role in the metabolic transition of tumors and the maintenance of phenotype of CSCs.

### The Role of Mitophagy in Tumor Progression

Mitophagy is a multi-pathway-regulated and multi-step process to degrade dysfunctional and damaged mitochondria. As a result, the role of mitophagy in tumor progression is very complex and depends on the type and stage of the tumor, as well as the specific mitophagy component molecules examined. Chourasia et al. (2015) reported that BNIP3 is most commonly deleted in patients with triple-negative breast cancer (TNBC). Similarly, BNIP3 functioned as a tumor suppressor in a MMTV-PyMT mouse mammary tumor model. Lack of Bnip3 caused an increment in tumor growth, invasiveness, and lung metastases. The role of BNIP3-dependent mitophagy, however, is controversial, partly depending on alternative pre-mRNA splicing of BNIP3. Normal cells express the full-length BNIP3, which promotes cell death. However, human adenocarcinoma cells preferentially express the truncated BNIP3 variant Bnip3Δex that lacks exon 3. The truncated Bnip3Δex3 isoform promotes tumor survival (Gang et al., 2015). It is also noteworthy that the move toward glycolytic metabolism upregulates the expression of the truncated Bnip3Δex3 isoform. Thus, BNIP3-mediated mitophagy may have a complex role in tumor growth and metastasis, depending on tumor type, alternative gene splicing regulation, and metabolic activity in cancer cells (Ferro et al., 2020).

Some studies suggested that PINK1 and PARK2 may function as tumor suppressors. Li C. et al. (2018) showed that expression of *PARK2* was associated with improved survival in patients with pancreatic cancer. Using a mutant *Kras*-driven spontaneous pancreatic tumorigenesis model, they found that genetic deletion of *Parkin* or *Pink1* promoted the development of pancreatic tumorigenesis. *PARK2* is a component in the p53 tumor suppressor pathway. Lack of PARK2 induced the Warburg effect with increased glycolysis and ROS production, potentially enhancing tumorigenesis (Zhang et al., 2011). FUNDC1 is a mitophagy receptor that mediates mitophagy in response to hypoxia. Li W. et al. (2019) showed that FUNDC1 was upregulated in human hepatocellular carcinoma (HCC) tissues. Using a hepatocyte-specific *Fundc1-*knockout mouse model, they demonstrated that *Fundc1* depletion enhanced the development and progression of HCC through the mitophagy-inflammasome pathway.

Overall, most mitophagy receptors or regulators are involved in cancer, but whether they function as tumor promoters or suppressors depends on tumor type and tumor microenvironment. Consistent with this dual role of mitophagy in tumors, mitophagy pathway proteins may be either overexpressed or downregulated in cancer (Table 1).

**TABLE 1:** The expression of mitophagy proteins in different cancer types.

Mitophagy protein	Expression levels in cancer	Cancer type	References
Parkin	Increased	Melanoma	Lee Y. S. et al., 2018
	Decreased	Lung cancer; breast cancer; glioma; pancreatic ductal adenocarcinoma; and colon cancer	Poulogiannis et al., 2010; Tay et al., 2010; D’Amico et al., 2015; Maugeri et al., 2015; Li C. et al., 2018
Pink1	Increased	Lung cancer; and esophageal squamous cell carcinoma	Yamashita et al., 2017; Liu et al., 2018
	Decreased	Colorectal cancer; glioblastoma; and pancreatic ductal adenocarcinoma	Poulogiannis et al., 2010; Agnihotri et al., 2016; Li C. et al., 2018
BNIP3	Increased	Prostate cancer; uveal melanoma; and renal carcinoma	Chen et al., 2010; Macher-Goeppinger et al., 2017; Jiang et al., 2018
	Decreased	Triple-negative breast cancer; and colorectal cancer	Bacon et al., 2007; Chourasia et al., 2015
BNIP3L	Increased	Pancreatic ductal adenocarcinoma	Humpton et al., 2019
	Decreased	Lung cancer	Sun et al., 2004
FUNDC1	Increased	Laryngeal cancer; cervical cancer; and breast cancer	Hou et al., 2017; Hui et al., 2019; Wu L. et al., 2019
	Decreased	Hepatocellular carcinoma	Li W. et al., 2019

## LncRNAs Emerge as New Epigenetic Regulators of Mitophagy in Cancers

### LncRNAs Deliver Epigenetic Messages Between Mitochondria and the Nucleus

Mitochondria and the nucleus contain distinct genomes. Mitochondria participate in crucial cellular processes involved in energy harvesting, intermediate metabolism, and apoptosis. Consequently, mitochondria must communicate and coordinate precisely with the nuclear genome to ensure proper cellular function and energy homeostasis (Barcena et al., 2018; English et al., 2020). This mitochondrial-nuclear crosstalk is coordinated by anterograde (from the nucleus to mitochondria) and retrograde (from mitochondria to nucleus) signals, ensuring that cells maintain homeostasis under basal conditions while enabling adaptation to various stressors (Noh et al., 2016; Quiros et al., 2016; Singh et al., 2017). In response to mitochondrial stressors, retrograde signals induce specific nuclear expression of proteins that migrate into the mitochondria to resolve these perturbations.

Recent studies suggest that lncRNAs may function as novel retrograde and anterograde signal molecules in this mitochondria-nuclear crosstalk (Dong et al., 2017; Zhao et al., 2018). LncRNAs are encoded not only from the nuclear genome but also from the mitochondrial genome (mtDNA). LncRNAs encoded by the mitochondrial genome can be exported into the cytoplasm and nucleus, where they can have pleiotropic interactions. Similarly, lncRNAs synthesized from the nuclear genome can be shuttled into the mitochondria, where they can modulate gene transcription or mitochondrial metabolism (Rackham et al., 2011; Noh et al., 2016).

The mitochondrial genome codes for 13 mRNAs, 22 tRNAs, and 2 rRNAs, producing proteins that participate in oxidative phosphorylation. In addition, several mtDNA-encoded lncRNAs have recently been characterized, including *lncND5*, *lncND6*, and *lncCyt B* (Rackham et al., 2011), and their expression is cell- and tissue-specific, suggesting an important role in the regulation of mitochondrial gene expression. Together, at least 18 mitochondria-associated ncRNAs have been identified, and they play critical roles in manipulating mitochondrial functions, altering metabolic reprogramming, mitochondrial genome transcription, stress signal transmission, and mitochondria-associated apoptosis (Zhao et al., 2018).

Using RNA-FISH with MitoTracker staining, Zhao et al. (2019) examined the mitochondrial localization of lncRNAs, and found that some nuclear genome-transcribed lncRNAs, like the oncogenic lncRNA *MALAT1*, were aberrantly translocated to the mitochondria of HCC cells; in normal liver cells, this lncRNA was found predominantly in the nucleus. *MALAT1* acts as an anterograde signal to regulate mitochondrial function in HepG2 cells. In contrast, the mitochondria-encoded lncRNA *lncCytB* acts as a retrograde signal in this mitochondria-nuclear crosstalk. This is in a sharp contrast to normal hepatic HL7702 cells, where *lncCytB* was primarily distributed in the mitochondria. Thus, aberrant shuttling of lncRNAs, whether nuclear-encoded or mitochondria-encoded, may be involved in the regulation of metabolic reprogramming in cancer cells.

### LncRNAs Regulate Mitochondrial Metabolic Reprogramming in Cancers

The role of lncRNAs as crucial regulators of the Warburg effect in cancer has recently been investigated (Shankaraiah et al., 2018; Cruz-Gil et al., 2020; Liu et al., 2021). LncRNA *EPB41L4A-AS1*, a downstream target of p53, was downregulated in several human cancers, including breast cancer, and its downregulation was associated with poor survival. Depletion of *EPB41L4A-AS1* expression induced the Warburg effect and increased aerobic glycolysis and glutamine metabolism through the HIF-1α pathway (Liao et al., 2019). *FILNC1* (FoxO-induced lncRNA-1), an energy stress-induced lncRNA, was downregulated in renal cancer. Under glucose starvation, *FILNC1*-depleted cells exhibited enhanced glucose uptake and lactate production, probably through the c-Myc network (Xiao et al., 2017). LncRNA *Ftx*, derived from the X-inactivation center on the X chromosome, was overexpressed in HCC tissues, and its expression was associated with malignant clinicopathological characteristics in HCC patients. *Ftx* was shown to promote the Warburg effect and enhance tumor progression through the PPARγ pathway (Li X. et al., 2018). *p21*, a well-known p53-inducible lncRNA, is also a key regulator of the Warburg effect and is essential for hypoxia-induced glycolysis (Yang et al., 2014). While these latter two lncRNAs are not located in the mitochondria, we believe that a wide variety of mitochondrial-associated lncRNAs play important roles in controlling energy production as well as mtDNA, providing potent therapeutic targets and diagnostic markers for cancer.

In hepatoma cells, the nuclear genome-encoded lncRNA *MALAT1* is abnormally transported into the mitochondria. Using an RNA reverse transcription-associated trap sequencing (RAT-seq) technology, Zhao et al. (2021) profiled the target binding sites of *MALAT1* in the mitochondrial genome. They showed that mitochondrial *MALAT1* lncRNA molecules were able to bind to multiple targets in mtDNA, including COX2, which encodes a subunit of the cytochrome c oxidase complex responsible for electron transfer in the mitochondrial respiratory chain. Depletion of the mitochondria-enriched MALAT1, either using shRNA or LwaCas13a-BN-MLS mitochondrial RNA targeting, altered mtDNA CpG methylation and induced multiple abnormalities in mitochondrial function and energy metabolism. Their findings suggest that lncRNA *MALAT1* may function as a novel nucleus-mitochondria epigenetic messenger. By shuttling between the nucleus and mitochondria, it epigenetically regulates mitochondrial metabolism in hepatoma cells.

### Interplay of Mitophagy and LncRNAs in the Maintenance of CSCs

Hypoxia is common inducer of mitophagy, and CSCs can adapt to the hypoxic tumor microenvironment through mitophagy and maintain their stemness. In addition, CSCs can use mitophagy to maintain their own self-renewal ability. The expression of HIFs after hypoxia can directly or indirectly stimulate the maintenance of stemness markers, such as OCT4, SOX2, etc (Zaarour et al., 2021). LncRNA XIST can increase the sensitivity of breast CSCs to chemotherapy drugs by regulating KMT2C (Huang et al., 2020). In liver cancer, lncRNA HDAC2 promotes the proliferation and self-renewal of liver CSCs by activating the Hedgehog signaling pathway (Wu J. et al., 2019).

Exosomal lncRNAs are key factors that mediate extracellular communication in the tumor microenvironment. DOCK9-as2 is an exosomal lncRNA, and its down-regulation reduces the proliferation, migration, invasion, epithelial-mesenchymal (EMT), and stemness of papillary thyroid carcinoma cells (Dai et al., 2020). In both *in vivo* and *in vitro* studies, Gao et al. (2021) demonstrated that exosomal lncRNA *UCA1* promoted self-renewal and differentiation of CD133 + cervical cancer cells through the micro-rRNA-122-5P/SOX2 axis. Silencing of *UCA1* reduced cell proliferation and invasion. Similarly, depletion of lncRNA *HotairM1* promotes self-renewal of CSCs through the HOXA1-Nanog loop (Li F. et al., 2020). These observations suggest that the crosstalk between hypoxia-driven HIFs, lncRNAs, and mitophagy plays a critical role in maintaining the stemness of CSCs.

### LncRNAs Are New Epigenetic Players in Mitophagy

LncRNAs are involved in the regulation of autophagy as well as autophagy-associated drug resistance. LncRNA *HULC* is highly upregulated in liver cancer and other malignancies. *HULC* overexpression is associated with poor overall survival and metastasis in cancers (Ding et al., 2019; Ghafouri-Fard et al., 2020). Knockdown of *HULC* suppresses autophagy and reduces cisplatin resistance by targeting downstream protein FoxM1 in drug-resistant gastric cancer cells (Xin et al., 2019). Another study reported that *HULC* triggered autophagy by stabilizing Sirt1, attenuating the sensitivity of HCC cells to chemotherapy (Xiong et al., 2017). Sun et al. (2017) found that lncRNA *HOTAIR* regulated cisplatin-resistance and autophagy by targeting Beclin-1, MDR, and P-GP in endometrial cancer cells. Finally, Liu C. et al. (2020) showed that lncRNA *RMST* was overexpressed in clinical glioma samples. *RMST* inhibited autophagy in glioma cells by inducing SUMO1 modification at K333 of FUS. SUMOylation of FUS promotes the degradation of ATG4D, a regulator of autophagy. More advances in the regulation of autophagy by lncRNAs in tumors are summarized in Table 2.

**TABLE 2:** Regulation of autophagy by LncRNA in different cancer types.

LncRNAs	Cancer type	Expression level in cancer	The effect of lncRNA on autophagy	The effect of lncRNA on chemoresistance	References
GBCDRlnc1	Gallbladder cancer	Increased	Promoted	Promoted	Cai et al., 2019
DCST1-AS1	Hepatocellular carcinoma	Increased	Promoted	**/**	Li J. et al., 2019
CCAT1	Hepatocellular carcinoma	Increased	Promoted	**/**	Guo et al., 2019
HAGLROS	Gastric cancer	Increased	Inhibited	**/**	Chen et al., 2018
NEAT1	Hepatocellular carcinoma	Increased	Promoted	Promoted	Li X. et al., 2020
NEAT1	Colorectal cancer	Increased	Promoted	Promoted	Liu F. et al., 2020
PVT1	Pancreatic ductal adenocarcinoma	Increased	Promoted	**/**	Huang et al., 2018
NBR2	Hepatocellular carcinoma	Decreased	Inhibited	**/**	Sheng et al., 2021
ATB	Hepatocellular carcinoma	Increased	Promoted	**/**	Wang C. Z. et al., 2019
DANCR	Hepatocellular carcinoma	Increased	Promoted	**/**	Wang X. et al., 2020
ZNNT1	Uveal melanoma	Decreased	Promoted	**/**	Li P. et al., 2020
SNHG7	Neuroblastoma			Promoted	Wang S. Y. et al., 2020
					

The crosstalk between autophagy and lncRNA may play a vital role in tumor progression. Using TCGA and CGGA databases, Xu et al. (Luan et al., 2019) analyzed 988 diffuse glioma patients and found that these patients were divided into two clusters with different prognostic outcomes based on the autophagy-related lncRNAs (ARLs) score. The ARLs signatures constructed by the authors showed good accuracy in predicting the prognosis of glioma patients. The ARL score was significantly elevated in the malignant subtype of glioma, and a high ARL score suggested a poor prognosis. A high ARL score indicated high infiltration of macrophages and neutrophils, thus serving as a promising prognostic biomarker for glioma patients. Using similar data analysis approaches, Li et al. (2021) found 11 ARLs in association with breast cancer prognosis. These 11 ARLs may become potential targets for autophagy-related targeted therapy.

**FIGURE 4:**
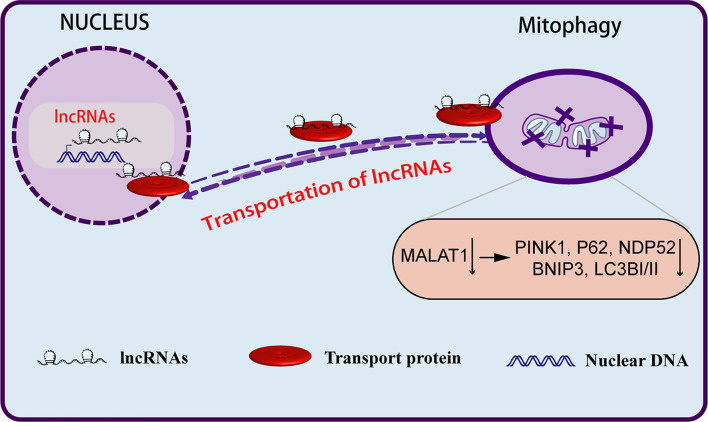
Long non-coding RNAs regulate the biological behavior of tumor cells through mitophagy. LncRNA MALAT1, after being transcribed from the nuclear genome, can be aberrantly transported to the mitochondria, where it epigenetically regulates mitochondrial functions and mitophagy. Depletion of MALAT1 downregulates mitophagy markers, particularly PINK1, P62, BNIP3, NDP52, and LC3.

The mechanism underlying the role of lncRNAs in the regulation of mitophagy remains unclear. Zhao et al. (2021) showed that the nuclear genome-encoded lncRNA *MALAT1* acts as a new epigenetic messenger by shuttling to the mitochondria, where it regulates mitophagy (Figure 4). Knockdown of *MALAT1* exhibited a significant decrease in mitophagy events. Mitophagy proteins, particularly PINK1, P62, NDP52, BNIP3, and LC3, were significantly downregulated in MALAT1-depleted HCC cells. This study proved for the first time that the lncRNA encoded by the nuclear genome might act as a new epigenetic player and regulate mitochondrial metabolism through mitophagy. In a second study, Xiang et al. (2021) observed the overexpression of PINT87, a peptide encoded by p53-induced transcript LINC-PINT, in senescent HCC cells. Overexpression of PINT87 inhibited mitophagy by directly binding to FOXM1 and blocking the transcription of PHB2, a crucial IMM receptor for Parkin-induced mitophagy.

## Application and Challenges of Modulating Mitophagy in Anti-Cancer Therapies

### Inhibition of Mitophagy to Enhance Sensitivity to Chemotherapy

Many anti-cancer drugs directly or indirectly cause damage to the mitochondria. Thus, the removal of dysfunctional mitochondria by mitophagy may alter their overall effectiveness (Bishop and Bradshaw, 2018). The induction of mitophagy during anti-tumor therapy may alter its cytotoxicity, leading to treatment resistance. ARIH1, an E3 Ub ligase belonging to the RING-between-RING (RBR) family, is overexpressed in many cancer cells. As a key regulator of mitophagy, ARIH1 activates the PINK1-dependent mitophagy that promotes the resistance to cisplatin chemotherapy (Villa et al., 2017). There is a tight regulatory crosstalk between apoptosis, mitophagy, endoplasmic reticulum stress, and mitochondrial dynamics/biogenesis in determining the fate of cells in chemotherapy (Abdrakhmanov et al., 2019). Stimulation of mitophagy with the protonophore carbonyl cyanide-*m* chlorophenylhydrazone (CCCP) inhibited the apoptosis induced by cisplatin, particularly at the high dose in HCT116 cells. On the other hand, inhibition of mitophagy enhanced the apoptotic response.

TMZ-POH, a novel conjugation analog of alkylating anticancer agent temozolomide, impaired mitophagy flux in non-small cell lung cancer (NSCLC) cells by inducing lysosomal dysfunction and hampering autophagosome-lysosome fusion. In radiotherapy, inhibition of mitophagy by TMZ-POH sensitizes cancer cells to irradiation-induced apoptosis (Chang et al., 2018). PINK1/Parkin-mediated mitophagy plays a critical role in hypoxia-induced radioresistance. Inhibition of this mitophagy pathway by a hypoxia-targeting p53 fusion protein, consisting of p53, TAT, and HIF-1α minimum oxygen-dependent degradation domain, sensitizes cancer cells in response to radiotherapy both *in vitro* and *in vivo* (Zheng et al., 2015). Collectively, these data support the pro-survival role of mitophagy in chemo/radiotherapy.

### Inhibition of Mitophagy to Adjust Drug Resistance in CSCs

Cancer stem cells are known to be drug-resistant. To study the role of mitophagy in CSC-mediated drug resistance, Yan et al. (2017) isolated CSCs carrying the CD133+/CD44+ marker from human colorectal cancer cells that were resistant to doxorubicin. Treatment with doxorubicin significantly upregulated the BNIP3L mitophagy pathway. Inhibition of this mitophagy pathway significantly enhanced the sensitivity to doxorubicin in CSCs. Thus, mitophagy may contribute to drug resistance in CSCs.

To examine the mechanisms underlying chemoresistance and its correlation with stemness, Naik et al. (2018) established cisplatin−resistant oral squamous cell carcinoma cells with CSC-like features. They found that mitophagy flux was significantly higher in cisplatin-resistant oral cancer cells than that in their parental counterparts, suggesting that mitophagy is responsible for chemoresistance in oral cancer. Inhibition of autophagy effectively downregulated the stemness and inhibited chemoresistance.

### Nucleus-Encoded LncRNAs as New Therapeutic Targets of Mitophagy

Long non-coding RNAs may promote tumor cell survival and reduce sensitivity of cancer cells to chemo- and radiotherapy by regulating mitophagy. Thus, targeting lncRNAs may be an appropriate approach to render cancer cells sensitive to chemotherapy. Cai et al. (2019) found that lncRNA GBCDRlnc1 (gallbladder cancer drug resistance-associated lncRNA1), a key regulator of chemotherapy resistance, is upregulated in gallbladder cancer. Depletion of this lncRNA inhibited autophagy and enhanced the sensitivity of gallbladder cancer cells to doxorubicin. Their findings established that targeting the chemoresistant driver GBCDRlnc1 might be an attractive therapeutic approach for the treatment of advanced gallbladder cancer.

Long non-coding RNA *H19* plays an important role in cell proliferation, metastasis, and chemotherapy resistance. Wang J. et al. (2019) showed that *H19* was significantly upregulated in breast cancer cell lines and tumor tissues that were resistant to tamoxifen treatment. Silencing H19 significantly inhibited autophagy and sensitized tumor cells to tamoxifen *in vitro* and *in vivo*. In contrast, overexpression of *H19* triggered autophagy in tamoxifen-sensitive cells and recapitulated the characteristics of tamoxifen resistance. Another study with lncRNA *KCNQ1OT1* in colon cancer patients found that *KCNQ1OT1* enhanced the chemotherapy resistance of colon cancer by activating autophagy through the miR-34a-Atg4B axis (Li Y. et al., 2019). Thus, targeting lncRNAs to regulate autophagy in tumors may be a promising therapeutic approach.

Disruption of the mitochondria-nuclear crosstalk network is a key event in many human diseases, including cancer (Mello et al., 2019). As critical regulatory components of this mitochondria-nuclear crosstalk, lncRNAs are also often dysregulated, including aberrant expression and shuttling between the nucleus and mitochondria. For example, the nuclear-encoded *MALAT1* can be shuttled to mitochondria, where it regulates the mitochondrial functions and mitophagy (Zhao et al., 2021). Thus, targeting a component of the dysregulated lncRNA-mitochondria-nuclear network may provide an ideal method to combat some malignancies. Novel targets include the nuclear expression of the lncRNA, nuclear-mitochondria shuttling, lncRNA binding to mitochondria, and downstream signals of the mitophagy pathway.

## Conclusion

Targeting mitophagy may present a new approach to develop anti-cancer therapies. However, currently we know very little about the precise mechanisms underlying the regulation of mitophagy in human tumors. Thus, it is critical to identify the role of mitophagy as well as the key regulatory components of each mitophagy pathway in tumor progression. More importantly, we must accurately define specific mitophagy regulators involved in radio- and chemoresistance in order to develop a precision medicine approach to target a specific component or a key regulator in mitophagy, rather than using inhibitors to target general mitophagy. A more comprehensive understanding of the key regulators of mitophagy in tumor progression may guide the development of novel therapeutics to treat some cancers (Bishop and Bradshaw, 2018). At the same time, it is also critical to understand the extent to which normal cells can tolerate these mitophagy inhibitors, so that drugs targeting mitophagy will not damage normal cells. More importantly, we should identify key regulatory factors that aberrantly regulate mitophagy in cancer cells to improve the precision of anti-tumor therapy.

The roles of mitochondria-associated lncRNAs in mitophagy are just beginning to be explored. As epigenetic regulatory factors, lncRNAs play important roles in intracellular environmental homeostasis, including mitophagy. Notably, some nucleus/mitochondria-shuttling lncRNAs are involved in the regulation of cancer metabolic reprogramming and the stemness maintenance of CSCs. After being transported to mitochondria, the nuclear-encoded lncRNAs not only epigenetically regulate mitochondrial metabolism, but also play a vital role in apoptosis and mitophagy. Therefore, precisely targeting mitophagy-related lncRNAs in cancer may become a very promising and attractive strategy for future tumor therapy with fewer toxic and side effects.

Since lncRNAs regulate mitophagy through multiple signaling pathways and mechanisms, more studies are needed before we target mitophagy-associated lncRNAs for precision medicine in cancer. For example, how are these nuclear lncRNAs transported into mitochondria? Can we interrupt this mitochondria-nuclear transportation to target mitophagy? What are the targets of these lncRNAs in mitochondria? How do these lncRNAs regulate mitophagy? A greater understanding of lncRNAs in this mitochondria-nuclear crosstalk network may help us develop novel lncRNA-based therapeutic approaches for malignancies that currently have few curative options.

## Author Contributions

YL wrote the manuscript. J-FH, JC, and WL supervised and funded the project. J-FH and AH edited the manuscript. All authors contributed to the article and approved the submitted version.

## Conflict of Interest

The authors declare that the research was conducted in the absence of any commercial or financial relationships that could be construed as a potential conflict of interest.

## Publisher’s Note

All claims expressed in this article are solely those of the authors and do not necessarily represent those of their affiliated organizations, or those of the publisher, the editors and the reviewers. Any product that may be evaluated in this article, or claim that may be made by its manufacturer, is not guaranteed or endorsed by the publisher.
